# Uterine natural killer cells ace an “A” in education: NKG2A sets up crucial functions at the maternal-fetal interface

**DOI:** 10.4049/jimmunol.2200384

**Published:** 2022-10-15

**Authors:** Francesco Colucci

**Affiliations:** 1 Department of Obstetrics & Gynaecology, University of Cambridge, National Institute for Health Research Cambridge Biomedical Research Centre, Cambridge CB2 0SW, UK; 2 University of Cambridge Centre for Trophoblast Research, Cambridge, UK

## Abstract

I argue here that reproduction was a driving force in the evolution of NK-cell education, which is set by interactions between inhibitory receptors and self MHC. Maternal lymphocytes also interact with allogeneic MHC on fetal trophoblast cells. How the maternal immune system accommodates the semi-allogeneic fetus is a fascinating question. But it may be the wrong question. Tissue lymphocytes, like uterine NK (uNK) cells, do not attack the mismatched fetus and its placenta. Instead, they help the local vasculature to accommodate changes necessary to nourish the fetus. Education of uNK cells, driven by the ancient CD94:NKG2A inhibitory receptor and self MHC, sets them up to deliver these key functions at the maternal-fetal interface. **/112**

## Beatles and Pink Floyd

**Figure 1:**
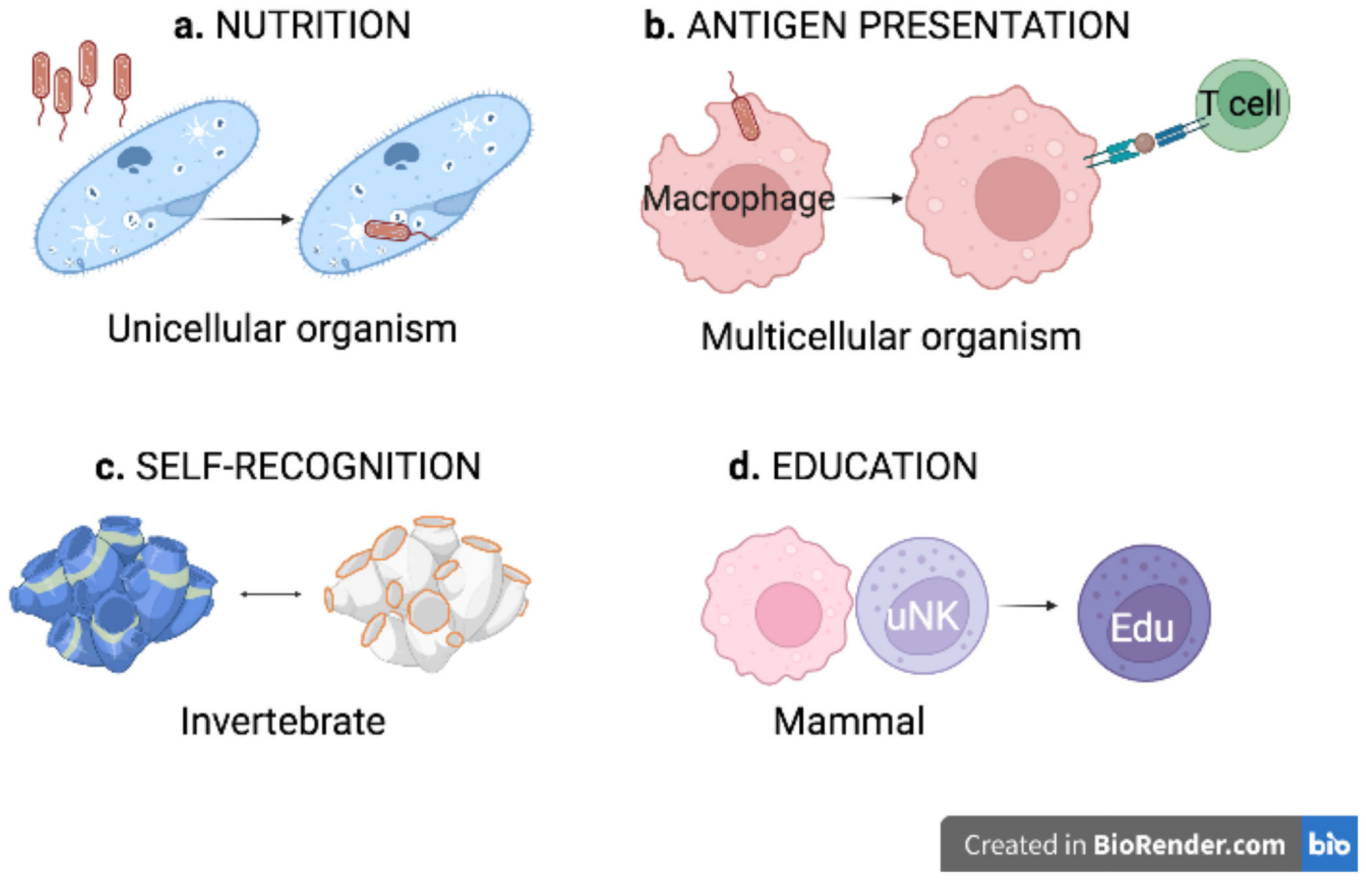
Repurposing of functions from primordial organisms to mammals. **a)** Nutrition in unicellular organisms was repurposed in **b)** phagocytosis to support immunity to pathogens through antigen presentation in multicellular organisms; **c)** Self-recognition in invertebrates may have evolved into **d)** education of mammalian uterine NK (uNK) cells. Created with BioRender.com

In his 2005 Pillars in Immunology paper, Lewis Lanier compared the counterintuitive concept of missing-self recognition by NK cells to the urban legend that the 1968 White Album hid information about a missing Beatle [1]. Playing the ‘Revolution #9’ track backwards, one could hear Lennon say: *‘Paul is dead, man, miss him, miss him’*. Klas Kärre’s 1984 idea that NK cells were activated by ‘missing’ MHC came in the wake of Doherty’s and Zinkernagel’s award-winning discovery of T-cell MHC restriction, and so it sounded just as backwards and hard to believe as the legend of the White Album. Nevertheless, Kärre’s theory became a classic [2], just like the urban legend. With Lanier’s and Kärre’s permission, I include Pink Floyd in this conversation. *‘We don’t need no education’* famously sung Pink Floyd in 1984. Today, >1,500 papers and seventeen years after its first description, NK-cell education still lacks a physiological *raison d’être*. There are even instances when uneducated NK cells outperform educated NK cells [3]. Shall we dismiss NK-cell education as a pure academic curiosity ? I don’t think so. Evidence is emerging that education is involved in HIV control [4], and in the outcomes of cell transplantation [5, 6], but it clearly did not evolve to help HIV escape immunity or surgeons perform better transplants. Here I argue that reproduction may have been a driving force in the evolution of NK-cell education. Could it have evolved from ancient pathways of self-recognition under the mighty selective pressure of reproduction? Placentation forced the immune system to come to terms with viviparity, that is the co-existence of two genetically distinct individuals in the womb. We think of placentation as the most significant event in the evolution of maternal tolerance of the fetus [7]. This may well be so, but that thought may guide us to seek misleading analogies with transplantation immunity [8]. Perhaps we ought to see beyond placentation and look towards simpler organisms to find ancestral self-recognition systems of NK-cell education. The illustrious precedent of Elie Metchnikoff’s discoveries in macrophage biology comes to mind [9]. The original nutritional function of unicellular organisms was repurposed into phagocytosis of macrophages and this supports immunity to pathogens through antigen presentation in multicellular organisms (Figure 1a-b). Some marine invertebrates use a recognition mechanism resembling NK-cell recognition that promotes reproduction [2] and this may have evolved into education of mammalian uterine NK (uNK) cells that sets up key functions for mammalian reproduction (Figure 1c-d).

## NK-cell education

MHC instructs NK cells to be tolerant of self and sets them up for function. This process is called NK-cell education [10, 11]. There is no strong evidence that it is required for any vital function. The questions we want to focus on in this paper are: does education help uNK cells to make babies grow? And if so, how? Does the MHC of the mother do the job or are maternal cells instructed by fetal MHC? And when fetuses do not express maternal MHC, are they at the mercy of uNK cells, like the *missing-self hypothesis* predicts?

Before we delve into the questions, we must ask ourselves what exactly is NK-cell education, and does it matter? NK-cell education is orchestrated through the recognition of self-MHC molecules by inhibitory NK receptors. The major receptors that mediate human NK-cell education are the ancient CD94:NKG2A receptor and the modern Killer-cell Immunoglobulin-Like Receptors (KIR). Mice also have CD94:NKG2A and functional homologues of KIRs, called Ly49 receptors. While CD94:NKG2A binds the ancient HLA-E in humans and Qa-1 in mice, KIR and Ly49 bind classical MHC class I molecules in the two species (HLA-A, -B, or - C in humans and H-2K or H-2D in mice). Little is known about the mechanisms that regulate NK-cell education, but we are learning its signalling pathways and membrane-proximal events [12–14]. We know that education of NK cells is genetically determined and depends on the inherited variants of polymorphic KIR and HLA class I genes. The same is true in mice too, where determinants are the Ly49 and MHC class I variants.

## The HLA-B→ HLA-E→ NKG2A pathway

About 50% of peripheral blood NK cells in humans or mice express CD94:NKG2A. The % of peripheral blood NK cells expressing any given inhibitory KIR (KIR-2DL1, -2DL2, -2DL3, -3DL1, -3DL2, -3DL3) varies from no cells to about 40%. While the percentages of KIR-expressing uNK cells vary slightly compared to peripheral blood NK cells, most (~95%) express CD94:NKG2A. What decides whether NK cells are educated by HLA-A, -B, -C→KIR or HLA-E→NKG2A? The choice between KIR or NKG2A is determined by an amino acid dimorphism at residue position -21 of the *HLA-*B leader sequence [15]. The expression of HLA-E depends on the availability of peptides derived from HLA-A, -B, or -C molecules. A methionine (M) makes for a productive HLA-B leader peptide that supports functional HLA-E expression. Adequate HLA-E expression educates CD94:NKG2A-expressing NK cells. Leader peptides with threonine (T) at this -21 position are poor peptides, resulting in low HLA-E expression and impaired NKG2A education. As a consequence, individuals with *M/M* and *M/T* genotypes educate NK cells through the HLA-B→HLA-E→NKG2A pathway, while individuals with *T/T* genotypes educate NK cells through the HLA-A, -B, -C→ KIR pathway [15].

## Education and inhibition

Inhibitory receptors trigger a signalling cascade that make NK cells refrain from responding to cells that express self MHC. This means that the same receptors that mediate NK-cell inhibition also mediate NK-cell education. So NK cells are set up by education to spring into action when they are disinhibited by ‘missing-self’ recognition. In other words, NK cells are educated to exert their functions when the opportunity arises. For blood NK cells, this is MHC downregulation in the case of virally infected cells or cancerous cells. In mismatched hematopoietic cell transplants, this is when donor NK-cell inhibitory receptors fail to engage the patient’s MHC [16]. In these instances, educated NK cells latch onto missing-self targets and kill them. How education sets uNK cells to deliver their functions is unknown because these functions differ from those of peripheral blood NK cells. While peripheral blood NK cells kill and produce pro-inflammatory cytokines, uNK cells are poor killers in physiological conditions, and instead act as ‘builders’ because they help remodel the uterine vasculature to sustain and support the growing feto-placental unit [17–20]. Education of uNK cells differs from education of peripheral blood NK cells, as we have shown [21]. Missing-self recognition for uNK cells is also unlikely because the system, at least in humans, seems to be geared up for the universal presence of an inhibitory signal. Virtually all (~95%) uNK cells express the inhibitory CD94:NKG2A receptor and its ligand HLA-E is expressed by both maternal and fetal trophoblast cells. Moreover, uNK cells interact also with allogeneic MHC expressed on the fetal trophoblast cells. Certain combinations of polymorphic KIR and HLA-C variants are linked to pregnancy complication and birthweight [22], but how does MHC influence uNK cell function? Let’s first take a look at the maternal-fetal interface.

## The maternal-fetal interface

Maternal immune cells and fetal cells come into contact at the maternal-fetal interface, where the blastocyst implants. It’s important to note that the fetus itself never makes contact with maternal immune cells, except for rare cells that enter the maternal circulation and generate microchimerism. It is the placental trophoblast cells that make direct contact with maternal immune cells. There are three different areas where maternal immune cells and trophoblast cells come into contact at the maternal-fetal interface [22]. One area of interface is between the maternal blood – where most lymphocytes are T and B cells – and two placental cell types, villous trophoblast and syncytiotrophoblast, both devoid of MHC molecules. Therefore, there is no molecular allo-recognition by maternal lymphocytes at this interface. The second interface is between maternal blood and extravillous trophoblast cells that have stripped the endothelial lining of the maternal blood vessels. Extravillous trophoblast cells do not express HLA-A or -B but express HLA-C and oligomorphic, non-classical MHC molecules HLA-E, and -G. The scope for allo-recognition by maternal T cells is narrow at this second interface. The third interface is deep into the maternal tissue. Some extravillous trophoblast cells invade into and through the decidua, up to the muscle layer. These trophoblast cells come into direct contact with tissue-resident immune cells, which are mainly uNK cells and macrophages. Here the interactions between polymorphic HLA-C molecules and maternal KIR generate large numbers of potential combinations. Some of these genetic combinations are linked to fetal growth and pregnancy disorders [22]. The current working hypothesis is that a balanced integration of inhibitory and activating signals is required for a physiological regulation of uNK cell function and healthy pregnancies. Those interactions that favour too much inhibition or activation of uNK cells are linked to pregnancy disorders [22]. Perhaps the best example is the correlation of inhibitory KIR with small babies and activating KIR with large babies [23]. In mice too, MHC influences fetal growth through interaction with uNK cells [24, 25].

## NK cells at the maternal-fetal interface

Human uNK cells are also known as decidual NK cells (dNK), because they are found in the uterine mucosa. Mouse uNK cells are found in the decidua and the muscle layer, and it is more appropriate to refer to them as uNK cells. We have recently determined the landscape of human dNK cells [26, 27] and described in detail the nomenclature of these cells in humans and mice [28]. Our first report on different mouse uNK subsets was in 2008 [29], around the time of the beginning of the research on innate lymphoid cells (ILC) [30] – NK cells were discovered in 1975 and Lymphoid Tissue inducer (LTi) cells in 1997 [31]. The discovery of ILC1, ILC2 and ILC3 has focused the attention of many immunologists on how tissue lymphocytes are part of our physiology, including skin, gut, lung and uterus [31]. While we are aware of different dNK subsets, their gene expression [32], phenotype and functional potential [26], the precise *in vivo* function of the different subsets is hard to determine [27]. For example, dNK cells produce factors, including GM-CSF, XCL-1, and MIP-1β that must affect the local micro-environment in ways we do not fully appreciate yet [26, 27]. Moreover, dNK cells can also respond to infectious challenges like Cytomegalovirus [33]. Recently, dNK-cell derived granulysin has been shown to be a key factor in cell-mediated immune responses to *Listeria monocytogenes*, a pathogen relevant to pregnancy complications in humans [34].

Human dNK cells can be grouped in 3 subpopulations of dNK1, dNK2 and dNK3, according to their pattern of gene or protein expression [26, 32]. Mouse uNK cells are sub-divided in CD49a^+^Eomes^+^ tissue resident (trNK) cells, CD49a^-^Eomes^+^ conventional (cNK) and CD49a^+^Eomes^-^ ILC1 cells [35, 36]. The frequency of these subsets varies during gestation. The largest population at mid-gestation is trNK cells, followed by cNK cells and a small ILC1 population [36]. uNK cells produce factors that help the local vasculature to accommodate changes necessary to nourish the foetus [27]. These changes are essential to enable the placenta to sustain healthy fetal growth. It is hard to compare the cell subsets in the two species. However, human dNK1 and mouse trNK cells, both phenotypically CD49a^+^Eomes^+^, do resemble each other and may be the key cells in the physiology of the uterus [27, 36, 37]. While we appreciate the amazing connection between maternal lymphocytes and fetal growth, the mechanisms are still unclear.

## uNK cell-education at the maternal-fetal interface

**Figure 2:**
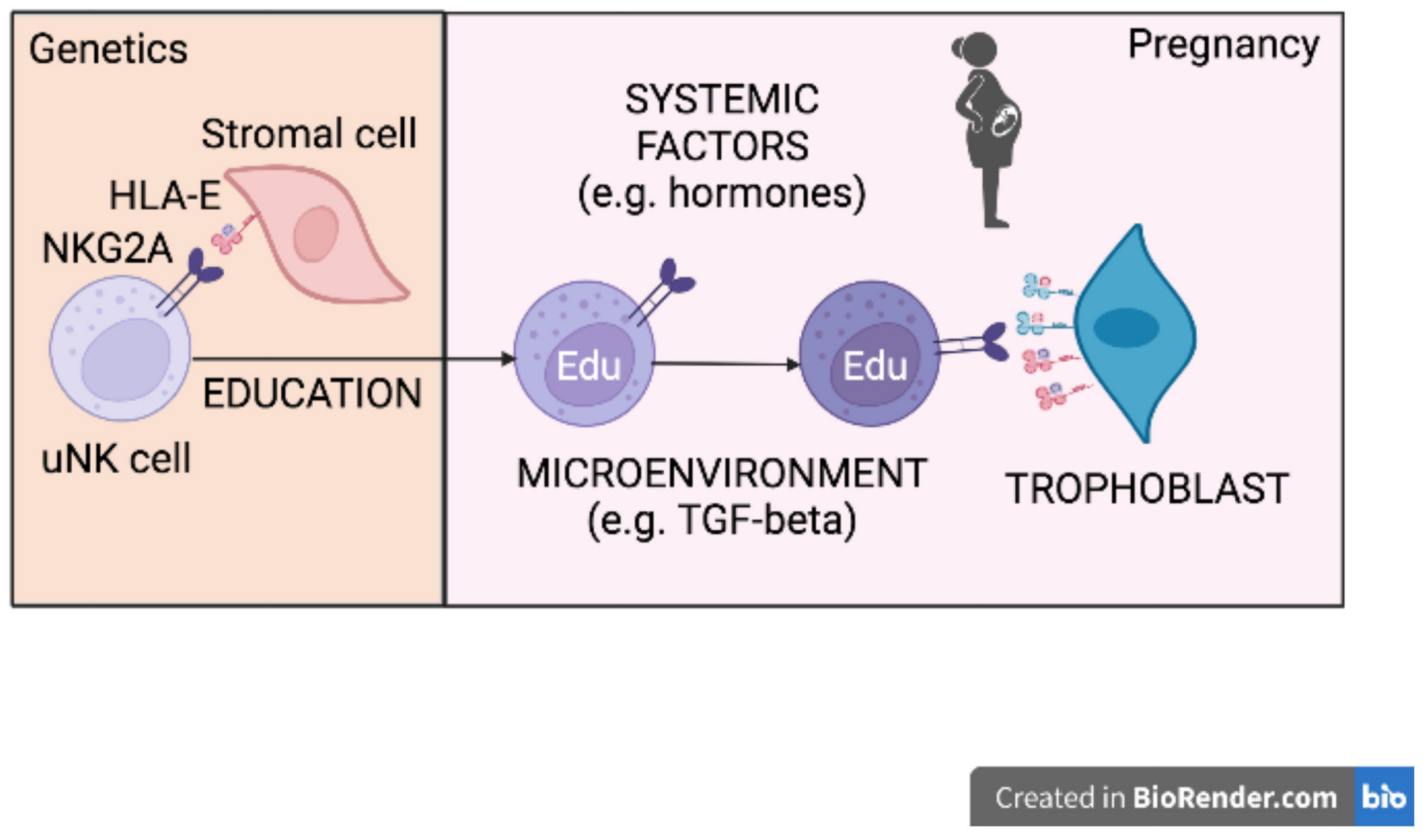
NKG2A cell education and inhibition at the maternal-fetal interface. Over 95% of uterine NK (uNK) cells express NKG2A. Prior to pregnancy, the HLA genetic make-up of the individual determines whether uNK cells are educated through NKG2A upon self-recognition of HLA-E on maternal cells in the uterus. Upon education, uNK cells (Edu) become functionally competent. During pregnancy, the reactivity of educated uNK cells is influenced by systemic factors, e.g. hormones, and the local tissue microenvironment, e.g. TGF-beta. One factor affecting uNK cell function is HLA-E expression by invading extravillous trophoblast. Because of the expression of HLA-G and the availability of the HLA-G leader peptide, extravillous trophoblast cells likely express more HLA-E than maternal cells at the maternal-fetal interface and may inhibit dNK cells, whether they are educated or not. dNK education and/or inhibition through HLA-C and KIR interaction are not depicted here for simplicity. Created with BioRender.com

uNK cells interact not only with trophoblast cells but also with decidual stromal cells and myeloid cells, that is maternal cells that express self MHC. Decidual stromal cells are fibroblasts conditioned by the tissue microenvironment and hormones that adapt to changes occurring during both the menstrual cycle and in pregnancy. While trophoblast can only interact with maternal uNK cells only during pregnancy, decidual stromal cells and myeloid cells interact with uNK cells at any time, during the cycle, and in pregnancy too. In my opinion, these cells are best placed to educate uNK cells (Figure 2). Designing an experimental setting to determine the role of uNK-cell education in human reproduction is extremely hard for obvious research limitations. Moreover, one has to contend with the extreme diversity of the KIR and HLA genes. A reductionistic approach is feasible in the mouse. The first ever KO mouse generated was the *β2m^-/-^* mouse [38, 39]. This model was key to test and prove the validity of the missing-self hypothesis [40]. We set out to address the questions mentioned before using various combinations of MHC-deficient and -sufficient female and male mice. Using these combinations, we could generate dams and offspring with or without MHC. In *β2m^-/-^* dams, in which NK-cell education cannot occur, uNK cells were hypofunctional. Because the uNK cells did not function properly, the uterine vasculature was not sufficiently remodelled. This most likely led to reduced blood flow to the fetuses. Consequently, the fetuses were growth-restricted even when they express their own, paternally inherited, MHC [41]. We concluded that uNK cell education is required for uNK cell function and optimal fetal growth. We also concluded that maternal and not fetal MHC drives uNK cell education.

In crosses where *β2m+/-* female mice were mated with *β2m-/-* male mice, we had an interesting opportunity. Maternal uNK cells were educated by self MHC and half the pups in these dams were expected to be MHC-deficient. The MHC-deficient pups would be exposed to missing-self recognition by the educated uNK cells, which may destroy them. However, we found that uNK cells in *β2m+/-* dams most likely did not attack their *β2m-/-* pups, because the ratio of their *β2m+/-* and *β2m-/-* pups was found to be exactly 50:50 over 11 litters and 84 pups [41]. This settles the case and shows the peaceful nature of uNK cells that do not attack missing-self targets. It is worth mentioning that the potential for *β2m-/-* trophoblast cells to activate uNK cells was probably intact because we found normal expression of ligands for uNK-cell activating receptors NKG2D and DNAM-1 [41].

But which receptor-ligand interactions are key to drive uNK cell education? We have recently found that CD94:NKG2A education is a determinant of uNK cell function. Genetic ablation of NKG2A in pregnant dams impaired the ability of their uNK cells to modulate the necessary vascular changes in the uterus during pregnancy, with consequent asymmetric fetal growth restriction and abnormal fetal brain development, which may eventually compromise offspring health [42]. In a genome-wide metanalysis of >150,000 pregnancies in European women we found that the *-21T* version of HLA-B (“poor NKG2A educator”) is associated with a 7% greater relative risk of developing the hypertensive disorder of pregnancy pre-eclampsia, which is often accompanied by fetal growth restriction. Taken together, we propose that the ancient, non-classical HLA-E pathway genetically determines NKG2A education [42]. However, this is likely not to be the only important pathways in all human populations, because the frequency of *-21 M/M* or *M/T HLA-B* genotypes varies across populations from as little as 9% in Australia to as much as 56% in South America [15].

## uNK cell education versus uNK cell inhibition

Because NK-cell inhibitory receptors mediate both education and inhibition, as we have discussed earlier, their relative contribution to the two functions is hard to determine. How can the same receptor set up a function through education, and then suppress it through inhibition? The solution, at least for peripheral blood NK cells, may be found in missing-self recognition, because NK cells spring into action when the ligand for that receptors is missing. An illustrative example is the role of NKG2A in NK-cell recognition of viral infections. HIV and CMV manipulate MHC expression to evade immune recognition, leading host cells to lower the expression of HLA-A and HLA-B. This avoids T cells. At the same time, HIV and CMV enhance the expression of HLA-C and HLA-E, which engage inhibitory KIR and NKG2A receptors on NK cells, thus escaping NK-cells too. This pattern of expression is similar to the unique pattern of human trophoblast described above. It is tempting to speculate that the immunological escape evolved by trophoblast cells might have functioned as a platform for viral escape. However, parallels between the biology of peripheral and uterine NK cells are inevitably confounded by the different nature of the function of the two populations of NK cells. Nevertheless, individuals with high HLA-E expression will have strong NKG2A education but may fail to control HIV infection because their NK cells may be strongly inhibited through NKG2A [4]. Similarly, uNK cells of women with high HLA-E expression and strong NKG2A education may be inhibited by even higher HLA-E expression on trophoblast cells. Trophoblast cells may express higher levels of HLA-E because they express also HLA-G, which provides optimal peptides for HLA-E expression (Figure 2). It is worth mentioning that the *-21M* or *-21T* genotype of the mother may not matter in this case, because the fetal trophoblast cells will likely express high levels of HLA-E regardless of maternal genetics.

Therefore, it is difficult to establish how education and inhibition of uNK cells through NKG2A play out in reproduction. Bearing in mind the power of monoclonal antibodies that ‘unleash’ the potential of lymphocytes in cancer patients by blocking checkpoint inhibitors, including NKG2A, we have designed experiments to try and disentangle the role of education from that of inhibition. HLA-E on tumour cells suppresses NKG2A-expressing NK and T cells in the tumour micro-environment and this can be reverted by blocking NKG2A [43]. We reasoned that if we let uNK cells be naturally educated by self MHC prior to pregnancy in wild-type dams, we could then block NKG2A with monoclonal antibodies during pregnancy and assess the effect on litter size and fetal growth. Assuming that acute interruption of NKG2A signalling does not interfere with NK-cell education but interferes with NK-cell inhibition, any effect of the treatment on pregnancy could be assigned to the lack of inhibition. We saw no significant effect on litter size or fetal growth, so we concluded that blocking NKG2A inhibition does not compromises healthy pregnancy, whereas the absence of NKG2A prior to pregnancy does [42].

## Conclusions

We are starting to crack the immunological code of pregnancy [17] and the CD94:NKG2A pathway emerges as a key piece of the puzzle. Phaenarete, Socrates’s mother, was a midwife. She would have been pleased to know that one of her son’s favourite mantras – *“Know yourself”* – matters to pregnancy outcomes because uterine uNK-cell education through self-recognition of maternal MHC helps uNK cells deliver necessary function for fetal growth [18, 32].
